# Exercise Snacks in Adults Living With Obesity: Protocol for a Randomized Feasibility Trial

**DOI:** 10.2196/100108

**Published:** 2026-06-12

**Authors:** Seth F McCarthy, Ella Harness, Noah Mulkewich, Roderick E Sandilands, Alexis Marcotte-Chénard, Hashim Islam, Douglas L Richards, Kenneth Madden, Joel Singer, Mary Jung, Martin J Gibala, Jonathan P Little

**Affiliations:** 1 School of Health and Exercise Sciences, Faculty of Health and Social Development, University of British Columbia, Okanagan Campus, 3333 University Way, Kelowna, BC, V1V 1V7, Canada, 1 250-807-9876; 2 Department of Kinesiology, Faculty of Science, McMaster University, Hamilton, ON, Canada; 3 Interdisciplinary School of Health, University of Quebec in Outasouais, Gatineau, QC, Canada; 4 Research Center on Aging, CIUSSS de l'Estrie - CHUS, Sherbrooke, QC, Canada; 5 Centre for Chronic Disease Prevention and Management, Faculty of Medicine, University of British Columbia, Okanagan Campus, Kelowna, BC, Canada; 6 Department of Medicine, McMaster University, Hamilton, ON, Canada; 7 Department of Medicine, Faculty of Medicine, University of British Columbia, Vancouver, BC, Canada; 8 School of Population and Public Health, Faculty of Medicine, University of British Columbia, Vancouver, BC, Canada

**Keywords:** obesity, physical activity, exercise, cardiorespiratory fitness, behavior counselling

## Abstract

**Background:**

Rates of obesity worldwide continue to increase and are associated with myriad health risks and socioeconomic burdens. Exercise is a traditional treatment for adults with obesity to increase physical activity levels, improve cardiorespiratory fitness, and reduce cardiometabolic risk. However, many people living with obesity do not engage in enough physical activity to achieve health benefits and often cite a perceived lack of time, lack of access to equipment, and stigma as barriers. “Exercise snacks” are short (~1 min) isolated bouts of vigorous exercise performed sporadically throughout the day that may be a viable strategy to improve fitness and cardiometabolic health. It is unknown whether exercise snacks are a feasible option in the real world for people living with obesity.

**Objective:**

This study aims to conduct a pilot randomized clinical trial (RCT) to determine the feasibility and preliminary efficacy of a 12-week smartphone app–supported exercise snacks intervention with behavior change counseling for improving cardiorespiratory fitness and other indices of cardiometabolic health in previously inactive adults living with obesity.

**Methods:**

A 2-site, parallel arm, RCT will be conducted in Kelowna and Hamilton, Canada. Eighty inactive adults living with obesity will be randomized to an exercise snacks or stretching/mobility exercise comparator group for 12 weeks. The former will complete 4 × 1-minute bouts of vigorous exercise at least 5 days per week, and the latter will perform mobility or stretching exercises using the same schedule. Interventions will be delivered through a customized smartphone mobile app and will be tailored to the participants’ schedules via onboarding counseling sessions, with ongoing telephone check-in call support at weeks 1, 2, 4, and 8. This pilot RCT will focus on feasibility as reflected by rates of recruitment, adherence, and dropout. We will also assess cardiorespiratory fitness, anthropometric markers, and routine blood health markers (eg, glucose, insulin) at baseline and postintervention.

**Results:**

This study is funded through the Heart & Stroke Foundation of Canada Grants-in-Aid program (2024/2025). Recruitment began on July 18, 2025 (Kelowna) and in November 2025 (Hamilton). As of March 31, 2026, we have enrolled 65 participants (University of British Columbia Okanagan, n=51; McMaster University, n=14). Data analysis will begin once all enrolled participants have completed the trial, and we expect to publish the results of the trial in winter 2027.

**Conclusions:**

This study will test an RCT protocol, provide evidence on the feasibility of exercise snacks in inactive adults living with obesity, and report preliminary effect size estimates for their ability to improve cardiometabolic health.

## Introduction

### Background

Obesity represents one of the largest health care challenges to Canadian society. In 2022, approximately 30% of Canadian adults were living with obesity [1], costing Canada approximately $28 billion annually in health care costs and lost productivity [2]. Obesity contributes to the development of a variety of cardiometabolic diseases such as cardiovascular disease, cancer, and type 2 diabetes [3]. While exercise represents a nonpharmacological treatment for many of the comorbidities associated with obesity, many adults living with obesity are insufficiently active [4,5]. Given the individual, societal, and economic burdens of obesity and its associated comorbidities, strategies targeting multiple aspects of cardiometabolic health are needed to help combat the obesity epidemic.

Chronic exercise is an established strategy to improve cardiorespiratory fitness [6-8], reduce blood pressure [9-11], as well as reduce adiposity and improve body composition [12-14] in adults living with overweight or obesity. Integrating small amounts of physical activity can have important implications as even modest improvements in fitness are associated with meaningful reductions in all-cause and cardiovascular disease mortality risk [15,16]. Despite these potential benefits, many people living with obesity are insufficiently active to achieve health benefits [4,5] and commonly cite a perceived lack of time and lack of access to equipment or facilities as barriers to physical activity [17]. Additionally, adults living with obesity face substantial stigma, which may limit motivation to participate in traditional exercise options (eg, fitness classes, exercising in public) [18,19]. Considering the deleterious health effects of obesity and low levels of physical activity in this population, there is an urgent need for time-efficient and accessible exercise strategies that can help individuals living with obesity become more physically active, improve their fitness, and reduce their risk of developing cardiometabolic diseases.

“Exercise snacks” are a practical and accessible form of physical activity that could improve cardiorespiratory fitness and cardiometabolic health in this population. Exercise snacks have been defined as isolated ≤1-minute bouts of vigorous exercise performed periodically throughout the day without the need for specialized equipment [20]. This approach to exercise may simultaneously promote engagement in vigorous physical activity and simultaneously interrupt sitting time [20,21]. Laboratory studies demonstrate that exercise snacks are well tolerated in inactive adults [22-24], including those living with obesity [25], and can improve cardiorespiratory fitness by approximately 4% to 5% compared to control or baseline [23,24]. Additionally, a laboratory-based study reported that on days exercise snacks are performed, adults living with obesity have lower postprandial plasma insulin and free-fatty acid levels compared to a day when they remain sedentary [25], highlighting the potential cardiometabolic benefits of brief bouts of exercise interspersed throughout the day. A recent 12-week randomized clinical trial (RCT) demonstrated that exercise snacks delivered through an online platform and mobile app were feasible to complete in the real world (ie, at home or work) with high enjoyment ratings in healthy (not living with obesity) but inactive adults [26]. The viability of exercise snacks to improve physical activity levels and cardiorespiratory fitness, while interrupting sedentary time and obesity-related risk factors, in people living with obesity is unknown. The proposed project will determine whether exercise snacks can be used as an approach to deliver the therapeutic benefits of physical activity in people living with obesity.

### Study Objectives

This study aims to conduct an RCT to determine the feasibility and preliminary efficacy of a 12-week smartphone app–supported exercise snacks intervention with behavior change counseling for improving cardiorespiratory fitness and other indices of cardiometabolic health in previously inactive adults living with obesity. Our specific aims are as follows:

1. Determine the proportion of individuals living with obesity who are eligible to participate, enroll in the trial, and are retained at 12-week postintervention testing
2. Measure adherence, cardiorespiratory fitness, plasma glucose, and insulin to inform effect size estimates for a future definitive RCT
3. Evaluate the acceptability of the intervention by identifying barriers and facilitators and the most effective and ineffective aspects of the intervention to optimize the delivery of a future RCT

## Methods

### Trial Design

**Figure 1.**
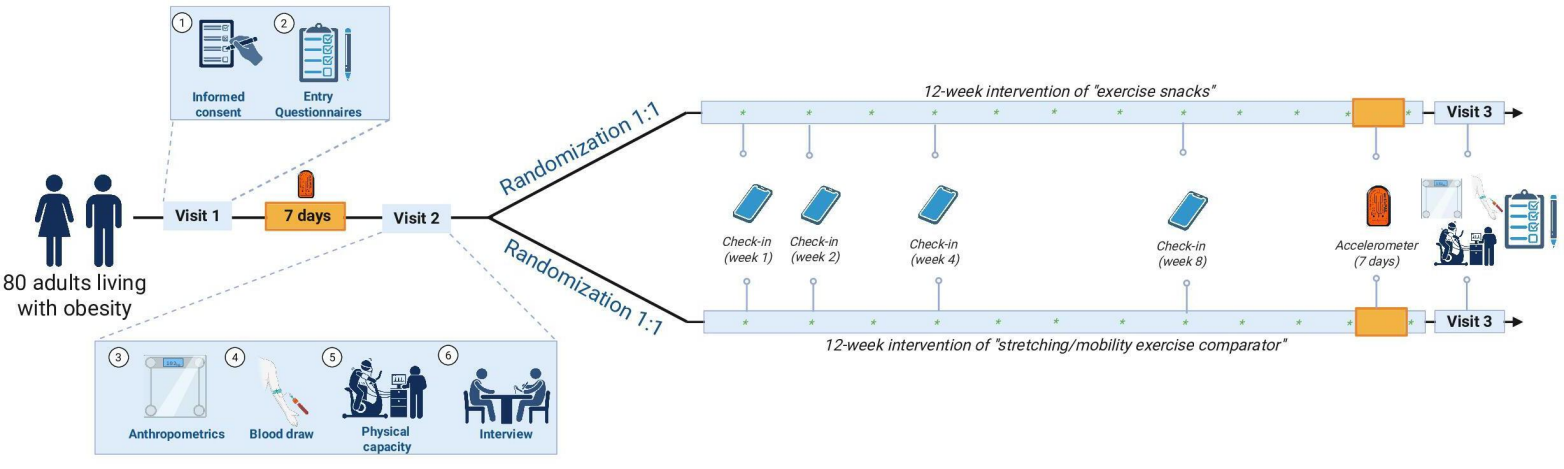
Study timeline. This pilot randomized clinical trial (RCT) will involve a parallel-arm design where participants are randomized into an exercise snacks or stretching/mobility exercise comparator group for 12 weeks. Eighty inactive adults living with obesity will be recruited from Kelowna, BC, and Hamilton, ON. Once deemed eligible via phone screening, participants will report to the laboratory for their first visit, where informed consent will be obtained, entry questionnaires completed, and eligibility confirmed. Participants will wear an accelerometer for 7 days and report back to the laboratory for the second visit, where all baseline measurements will be conducted; the participants will be randomized, and they will undergo a behavior counseling session. Afterward, the participants will begin the 12-week intervention and have check-in calls at weeks 1, 2, 4, and 8. Participants will wear the accelerometer for 7 days during the last week of the intervention and then report back to the laboratory for the postintervention testing, where all outcomes will be reassessed.

This pilot RCT will involve a parallel-arm design where participants are randomized (1:1 allocation ratio stratified by sex and site) into an exercise snacks or stretching/mobility exercise comparator group for 12 weeks (Figure 1). Target enrollment is 80 participants recruited from Kelowna, BC (University of British Columbia [UBC] Okanagan), and Hamilton, ON (McMaster University), and their surrounding areas using online advertisements supported by a recruitment service agency (Wayturn, Mariefred, Sweden). At each site, potential participants’ contact information is provided to the research team, which will initiate contact to schedule a screening call to determine eligibility before booking the first baseline study visit. Interventions will be delivered at home or work via a custom study mobile app and individualized via onboarding telephone exercise counseling sessions to develop an action plan for incorporating exercise snacks or stretching exercises into participants’ daily routines. Primary outcomes focused on feasibility will be assessed over the course of the intervention, and secondary outcomes focused on efficacy will be assessed at baseline and following the 12-week intervention. A flow diagram for the trial is presented in Figure 1.

### Ethical Considerations

During visit 1, informed consent will be obtained (by the lead trainee at each site) before any trial-related procedures are carried out, eligibility will be confirmed, and participants will be familiarized with the study procedures. During visits 2 and 3, secondary outcomes will be assessed. The protocol was approved by the UBC Clinical Research Ethics Board (H21-03417; version 10) and the Hamilton Integrated Research Ethics Board (19021; version 4) and was registered on ClinicalTrials.gov (NCT06924346) on July 17, 2025, before any participants were enrolled. This draft is version 5 of this protocol. Should a need arise that requires an amendment to the study protocol, the research team will gather to discuss the potential changes. If substantial amendments are required, they will be submitted to the site-specific ethics boards, and once approved, they will be implemented. The study is sponsored by the UBC Okanagan and funded by the Heart & Stroke Foundation of Canada (G-24‐0036465). The sponsor and the funder have no influence on study design, collection, management, analysis, and interpretation of data, writing of the report, and the decision to submit for publication. The data will be collected and stored in accordance with UBC Clinical Research Ethics Board and Hamilton Integrated Research Ethics Board policies. All data will be entered electronically, and files will be stored on secure servers at both sites for 5 years according to the policies outlined in each site’s respective ethics board. The data management team will consist of the principal investigators (PIs) at each site, the lead trainee at each site, and the trial statistician, who will meet monthly. The PI will be responsible for the integrity of the data stored on a secured Microsoft Teams channel, with the lead trainee and trial statistician responsible for database cleaning and locking. Only members of the research team will have access to the data during and after the completion of the study.

### Participants and Eligibility Criteria

Eligible participants will be living with obesity, currently inactive, and aged between 35 and 64 years. Potentially eligible participants will be contacted via phone or email to complete the eligibility screening, which includes a medical history, self-reported anthropometric measurements, current physical activity behavior, and details of current medications. Once participants have consented to participate, they will be assigned a deidentified code, and all data will be linked to their deidentified code rather than identifying information (eg, name, date of birth, email address). Informed consent forms and any other data collection sheets that contain identifying information will be stored separately from deidentified data.

**Figure 2.**
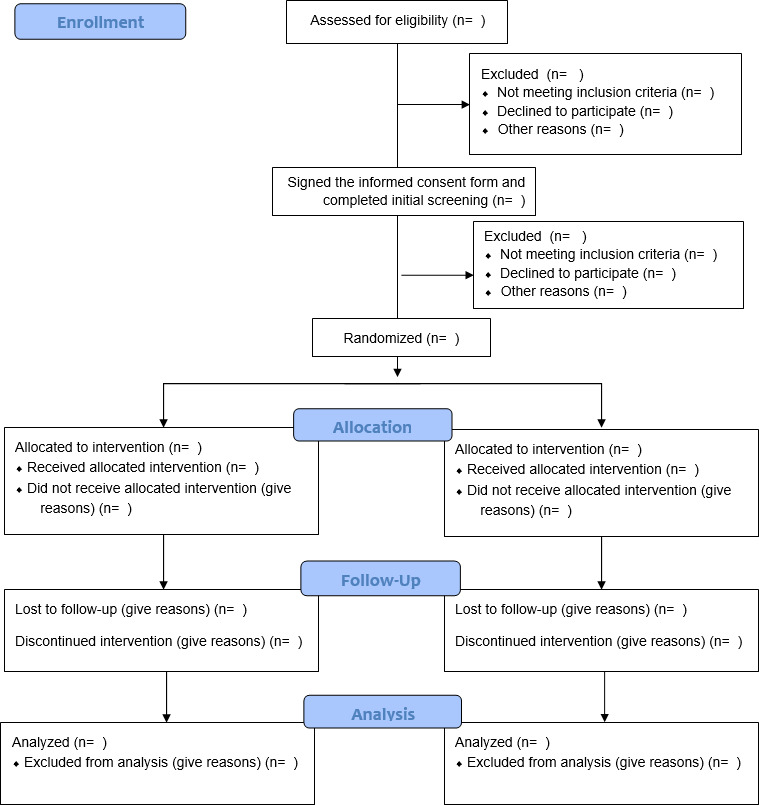
Trial flow diagram that will be used to determine the number of participants expressing interest, the number of participants who sign the informed consent form and complete the initial screening, the number of participants randomized to each group, the number of participants who complete the intervention, as well as the number of participants who are included in the final analysis. At all steps, the number of participants who are excluded or withdraw from the study will be recorded.

There are no formal guidelines for determining sample size for a pilot trial, but published simulations suggest that 35 participants per group are appropriate for estimating the pooled SD for normally distributed variables to allow for accurate effect size estimates on outcomes [27]. This sample size is in line with the median sample size for pilot (n=30) and feasibility (n=36) trials in a published audit of registered pilot or feasibility trials [28]. In line with this, we will aim to recruit 40 participants per group (80 participants in total). Detailed inclusion and exclusion criteria are described in Textbox 1; participants will first be screened for these criteria during a screening phone call followed by confirmation during the first laboratory visit (Figure 2).

Textbox 1. **Detailed inclusion and exclusion criteria.**

**Inclusion criteria**

- Aged 35‐64 years
- Engaging in ≤150 minutes of moderate-vigorous physical activity per week assessed using the Canadian Society for Exercise Physiology (CSEP) Get Active Questionnaire.
- BMI ≥30 kg/m^2^ (≥27.5 for participants who self-identify as being of Asian or Southeast Asian origin).
- Waist circumference ≥88 cm for female and 102 cm for male participants (≥80 cm for female and ≥90 cm for male participants who self-identify as being of Asian or Southeast Asian origin).
- Not currently diagnosed with a cardiometabolic disease (eg, coronary artery disease, stroke, diabetes, nonalcoholic fatty liver disease).
- Taking ≤2 commonly prescribed medications for the prevention of cardiometabolic diseases (eg, statins, antihypertensives, glucagon-like peptide-1 receptor agonists).

- Cleared to engage in physical activity using the CSEP Get Active Questionnaire and, if applicable, consultation with a health care provider or certified exercise physiologist.
- Not a current smoker.
- Access to a computer, tablet, or smartphone for intervention delivery and tracking.
- Able to read and write in English.

**Exclusion criteria**

- A chronic musculoskeletal condition or recent (within 2 years) cardiovascular event preventing participation in exercise.
- Lack of internet access.
- Angina upon exertion assessed by the Rose Angina Questionnaire.
- Prescribed beta-blockers that can compromise the validity of heart rate measurements during exercise testing.
- Have a scheduled surgical procedure within the next 3‐4 months that would prevent exercise participation.
- Currently participating in another clinical trial that interferes with the study procedures.
- Currently pregnant or planning on becoming pregnant during the intervention (ie, within the next 4 months).

### Randomization and Blinding

The randomization of participants will occur during the second baseline testing visit after the collection of baseline measures. Participants will be randomized using a 1:1 allocation ratio with variable permuted block sizes (stratified by sex and site) through the web-based platform REDCap (Research Electronic Data Capture) tools hosted at the UBC (Research Electronic Data Capture, Nashville, USA) [29,30]. A researcher not involved in the project created the randomization sequence. When a new participant completes baseline testing and is ready for randomization, the participant’s sex and site will be entered by the researcher into REDCap, which will display the randomization (exercise snacks, stretching exercise comparator) for that participant. Due to the nature of the intervention, blinding of the participants or the researchers delivering the intervention is not possible.

### Interventions

Participants in both groups will be instructed to complete at least 4 × 1-minute exercise bouts periodically throughout the day on at least 5 days per week (ie, a minimum prescription of 20 minutes of vigorous activity via 1-minute exercise snacks per week). This aligns with recent updates to the World Health Organization Guidelines on Physical Activity and Sedentary Behavior, which recognize the health benefits of short activity bouts [31], and falls within Canada’s 24-hour Movement Guidelines for Adults recommendation to break up long periods of sitting as much as possible [32]. The timing of exercise bouts will be tailored to participants’ daily schedules, and the complexity of movements will be customized to participants’ preferences and abilities. The interventions will be delivered through the Seven Movements app (Seven Movements), which will provide daily reminders to perform the exercise bouts. Seven Movements developed this app for the study but had no involvement in the study design and will not be involved in the analysis or reporting of the results. Notifications through the app will be customized to each participant’s schedule during the onboarding session completed at the baseline visit and adjusted during subsequent telephone check-ins if needed. Instructional videos prepared by the research team will be provided for each movement to ensure the participants are performing the exercise correctly and safely. The videos will provide brief (10 s) instructions and show individuals completing the exercises along with a 1-minute timer with brief audio encouragement throughout. In the app, participants will be able to log the number of daily exercise bouts completed, as well as the corresponding perceived effort (Borg CR-10 scale [33]) and enjoyment (Exercise Enjoyment Scale [34]).

### Exercise Snacks Group

Exercises for the exercise snacks group are designed to increase heart rate over the course of 1 minute and include running on the spot, speed squat, change of direction run, box run, shadow box chair squat, jumping jack, stair climb, twisty jack, side shuffle, and alternating lunge. Participants in this group will be instructed to complete the 1-minute exercise bouts at an intensity that would elicit a Borg Category-Ratio-10 rating of perceived exertion (RPE) value of ≥7. During visit 2, participants will complete 2 familiarization exercises to ensure they understand the required intensity to elicit an RPE value of ≥7.

### Stretching Exercise Comparator Group

Exercises for the stretching/mobility exercise comparator group are mobility and stretching-based and designed not to raise heart rate. Movements include shoulder stretch, wall-chest stretch, quadriceps stretch, wall-calf stretch, cat-cow, seated glute stretch, hamstring stretch, high-lunge arm-raise, side bend, and kneeling hip stretch. Participants in this group will be instructed to complete the 1-minute exercise bouts at an intensity that would elicit a BORG CR-10 RPE value of 2‐3. This was selected as the most appropriate control group in attempts to balance randomization expectations, retention, and ethical considerations compared to a true “no exercise” control.

### Onboarding and Check-in Calls

After participants are randomized during baseline visit 2, they will meet with a research assistant for an onboarding session to further individualize the intervention and promote adherence. During the onboarding session, each participant will be provided with more detailed instructions on how to complete the exercise bouts using the smartphone app and help customize the exercise bouts prescription to fit their daily schedules (eg, choosing days of the week and times to do the exercise bouts). Using motivational interviewing techniques designed to overcome anticipated barriers and increase adherence [35-37], the research assistants facilitating these sessions will use a standardized brief action planning [38,39] interview guide to codevelop individual plans with each participant.

Participants will meet with research assistants for telephone check-in calls at the end of weeks 1, 2, 4, and 8. The check-in calls at the end of weeks 1 and 2 will focus on troubleshooting any issues experienced with the smartphone app, providing modifications for exercises, and checking if participants’ adherence and RPE match their group’s prescription. If participants request modifications for exercises, these instances will be recorded in a check-in call summary document that summarizes the main points discussed with the participant. Check-in calls at weeks 4 and 8 will be used to assess progress and goals developed during the onboarding session, provide continued support throughout the trial, and make any modifications to the exercise plan.

### Outcome Measures

Primary and secondary outcome measures are listed in Textbox 2 and then described in full detail in the text. Primary outcomes are all related to feasibility and collected over the duration of the intervention. Secondary outcomes are related preliminary to efficacy and are collected at baseline and postintervention.

Textbox 2. **Primary and secondary outcomes.**

**Primary outcomes**

- The number of individuals eligible to participate in the trial, the number of participants approached, and reasoning for not joining the study.
- The number of eligible participants who enroll in the study.
- The number and percentage of participants retained after the 12-week intervention, as well as the number of participants who drop-out and reasons.
- The number of 1-minute exercise bouts performed during the 12-week intervention.

**Secondary outcomes**

- Cardiorespiratory fitness estimated from walking treadmill test.
- Lower body muscular endurance assessed using sit-to-stand test.
- Upper body maximal strength assessed using hand-grip dynamometer.
- Waist circumference.
- Weight.
- BMI.
- Fasting plasma glucose.
- Fasting plasma insulin.
- Systolic blood pressure.
- Diastolic blood pressure.
- Physical activity estimated using accelerometry.

Feasibility outcomes will include the number of participants who are eligible to participate, the number of participants who enroll, the number of participants who are retained at 12-week postintervention testing, as well as the number of 1-minute exercise bouts completed during the intervention. The proportion of participants contacted, screened, and enrolled in the intervention will be recorded, as well as the number of eligible participants who chose not to participate (and reasons why). Adherence will be determined by summing the total number of 1-minute exercise bouts completed during the intervention. The primary adherence outcome will be app-reported adherence, and this will be supplemented (where needed) with participant self-report (via weekly REDCap surveys) for participants who choose to perform movement breaks outside of the app. We will define high, moderate, and low adherence as completing >70%, 50% to 69%, or <50% of the prescribed intervention, respectively. Feasibility is defined as >70% of the participants completing at least 67% of the exercise bouts (ie, >13 weekly bouts) on at least 8 out of 12 weeks.

A modified Balke walking treadmill test will be used to estimate cardiorespiratory fitness [40-42]. To begin, participants will complete a 3-minute warm-up at 3.2 km/h and 0% incline on a motorized treadmill. Upon the completion of the warm-up, the test will begin, and the speed will be increased to 5.3 km/h where it will remain for the remainder of the test, with 1% increases in grade every minute until volitional fatigue. Participants will be familiarized with this test during the first baseline testing visit, and if the test duration is ≤7 minutes, ≥13 minutes, or ≥15 minutes, then the speed for all subsequent tests (visits 2 and 3) will be adjusted to 4.5, 6.1, or 6.9 km/h, respectively. The speed and maximal grade attained on the test will be used to estimate cardiorespiratory fitness using the calculation below [43].


$${VO}_{2\max}\;(mL\;kg^{-1}\;min^{-1})=speed\;(m\;min^{-1})\times \left(0.17+(fractional\;grade\times 0.79)\right)+3.5$$

Lower body muscular endurance will be assessed using the 30-second sit-to-stand test. Participants will start in a seated position with their feet flat on the floor at shoulder width and their hands on their shoulders. Participants will be instructed to fully stand up and sit down as many times as possible within the 30-second period. A stopwatch will be used to record the time, and the number of repetitions will be counted by 2 researchers. If the participant has completed a full stand from the sitting position when the time has expired, the final repetition will be counted in the total.

Upper body maximal strength will be assessed using a hand-grip dynamometer. The grip on the dynamometer will be adjusted so that the participant can comfortably grip the device and their fingers comfortably fit under the handle at the second knuckle. Participants will be asked to hold the dynamometer in line with the forearm, at thigh height, away from the body. Grip strength will be measured twice on both hands, alternating after each attempt. If a difference of more than 5 kg is recorded between 2 trials (for the same hand), a third trial will be conducted. The maximum score for each hand measured to the nearest kilogram will be recorded and combined for a total score.

Waist circumference will be measured using a tape measure (calibrated at regular intervals) at the level of the superior lateral aspect of the iliac crest. Participants will be asked to lift their shirts to expose their abdomen and landmark the superior lateral aspect of the iliac crest. The assessor will place the tape measure around the landmarked location, and after ensuring it is level around the abdomen, the measurement to the nearest 0.5 cm at the end of expiration will be recorded. The average of 2 measurements will be recorded. If participants are not comfortable exposing their abdomen, the measurement will be conducted over the shirt and repeated at post-testing. Weight will be measured to the nearest 0.1 kg using a mechanical scale, and participants will be asked to remove their shoes, remove any baggy clothing, and empty their pockets before stepping on the scale. Height will be measured to the nearest millimeter using a stadiometer using the same instructions as the weight measurement. Height and weight will be used to calculate BMI, which will be expressed as weight in kilograms divided by height in meters squared.

Fasted blood samples will be obtained from an antecubital vein for the measurement of plasma glucose and plasma insulin. Once the samples have been collected, they will be centrifuged, and plasma aliquots will be stored at −80 °C. Plasma glucose and insulin will be analyzed using commercially available assays (Chrystal Chem). Homeostatic model assessment of insulin resistance (HOMA-IR) will be calculated using the calculation below [44].


$$HOMA\text{-}IR=\frac{fasting\;insulin\;(\mu U/mL)\times fasting\;glucose\;(mmol/L)}{22.5}$$

Blood pressure will be measured on the left arm using an automated blood pressure monitor. Prior to the measurement, participants are encouraged to use the bathroom and void their bladder. Blood pressure will be measured after 5 minutes of quiet seated rest. Three measurements will be taken with 1-minute rest in between measurements, and additional measurements will be taken, if necessary, until 3 measurements are obtained where systolic blood pressure is ≤5 mm Hg of one another. During the 5 minutes of quiet seated rest and while measurements are being conducted, the participant will be asked to sit motionless with their feet together, back against the backrest of the chair, and their left arm resting on the table beside them.

Physical activity will be assessed using activPAL4 accelerometers (Pal Technologies Ltd). The devices will be initialized using PALconnect (v9.1.2.168) to collect triaxial acceleration data at 20 Hz with a dynamic range of ±4 g and will be worn on the midline anterior aspect of the nondominant thigh for 7 consecutive days at baseline and week 12. The devices will be waterproofed and secured with a transparent film dressing, and participants will be instructed to remove and reattach the device only if the adhesive loosens. Upon retrieval, raw .datx files will be downloaded using PALconnect (v9.1.2.168), processed using PALbatch (v9.1.0.77), and exported as uncompressed, uncalibrated .csv files in g format to enable further processing. Raw acceleration data will be processed using the open-source R package GGIR (v3.2.9) using default signal-processing procedures, including automatic calibration using local gravity [45], nonwear detection, identification of sustained abnormal acceleration, and calculation of 5-second ENMO (Euclidean Norm Minus One) values [46]. From GGIR day-summary outputs, average acceleration (mg), intensity gradient, most active “x” minutes metrics ranging from M1 to M10, and time accumulated in 50 mg incremental acceleration bins from 0 to >400 mg will be extracted to characterize overall activity volume and the distribution of activity intensity across the 24-hour day [47,48]. Participants will be included if they provide at least 3 valid days of data, defined by ≥75% device alignment [49], ≥16 hours of valid wear time, and a calibration error <10 mg [46].

### Communication of Findings

As we have registered the trial on ClinicalTrials.gov, once data collection is complete, we will report the results on ClinicalTrials.gov, including a flow chart of study recruitment and dropout, baseline characteristics of participants, and primary and secondary outcomes. We will share the findings from this study in the form of abstracts, as well as oral and poster presentations at national and international conferences related to exercise and physical activity (eg, Canadian Society for Exercise Physiology or American College of Sports Medicine annual meetings). We also intend to publish our findings in peer-reviewed journals. Three months following the completion of data collection, the research team will create a combined (both sites) deidentified dataset, including a codebook with the variables outlined in this protocol that will be used for statistical analysis. This deidentified codebook will be stored at UBC Okanagan on a secured Microsoft Teams channel with access controlled to study personnel listed on the ethics approval. The PI will share a deidentified dataset with outside investigators upon reasonable request according to the policies in the approved UBC Clinical Research Ethics Board protocol.

### Study Withdrawal

Participants will be instructed during the first study visit that they may withdraw from the study at any point and can do so by communicating to the research team (using any method) that they would like to withdraw. The data collected up to the point of withdrawal will be included in the analysis, and the reason for withdrawal will be recorded.

### Safety and Adverse Event Reporting

Prior to exercise testing, participants will be provided with descriptions of the common (ie, muscle fatigue, increased breathing rate, increased heart rate) and uncommon (ie, chest pain, difficulty breathing, light-headedness or dizziness) musculoskeletal and cardiovascular symptoms experienced during exercise so that participants are aware of what is normal and what is not. Participants will be instructed to notify the researchers immediately if they experience uncommon or exaggerated symptoms, and testing will be stopped immediately. In this scenario, the participant will be monitored in the laboratory for at least 20 minutes, with blood pressure and heart rate measurements taken every 5 minutes. Should symptoms worsen following the cessation of exercise, emergency services will be contacted. Should a participant experience any uncommon or exaggerated response to exercise, it will be recommended that they visit the local emergency room, consult with their family physician, or discuss with the study clinician.

All adverse events will be reported and assigned a severity or grading by the PI from each site responsible for the participant. The assignment of causality will be made by an independent clinician. All nonserious adverse events will be recorded from the point of consent until the completion of the final visit. PIs will report serious adverse events to their sponsor within 48 hours of the local site becoming aware of the event. In the case of multiple (≥2) musculoskeletal or cardiovascular instances that would require serious adverse event reporting, the trial steering committee (JPL, MJG, and SFM) would consult the study clinicians (KM and DLR) to determine if the trial needed to be stopped or modifications to the inclusion or exclusion criteria or protocol were needed. PIs from the 2 sites will be responsible for stopping the trial if required.

### Statistical Analysis

The proportion of eligible participants who consent to participate will be presented by site and overall, along with the proportions in each intervention group completing postintervention testing and the reasons for withdrawal. Descriptive characteristics and outcomes will be summarized overall and by intervention group as mean (SD) for normally distributed continuous variables, median (IQR) for nonnormally distributed continuous variables, and number (percentage) for categorical variables. The exit questionnaire will include open-ended questions to help us explore the barriers and facilitators, assessing which elements of the intervention are most effective for participants. Although inferential statistics are not necessary for pilot trials, we will perform exploratory analyses on outcomes in order to describe between-group treatment effects at 12 weeks with 95% CIs and calculate preliminary effect sizes. Continuous dependent variables will be analyzed using constrained baseline longitudinal analysis via linear mixed models with group and time as fixed effects and participant and study site as random effects [50]. The between-group effect estimate at 12 weeks will be the primary outcome of interest in these exploratory analyses. The data will be analyzed based on the intention-to-treat principle, with the assumption of data missing at random. Exploratory sensitivity analyses based on participants with high, moderate, or low adherence will be explored if appropriate. No interim analysis will be conducted. The statistical analysis plan was posted to the Open Science Framework on April 30, 2026 [51].

## Results

This study is funded through the Heart & Stroke Foundation of Canada Grants-in-Aid program (2024/2025). Recruitment began on July 18, 2025 (following trial registration), at UBC Okanagan and in November 2025 at McMaster University. As of March 31, 2026, we have enrolled 65 participants (UBC, n=51; McMaster University, n=14).

## Discussion

### Rationale, Significance, and Limitations

Obesity rates around the world [52] and in Canada [2] have continued to rise at alarming rates over the last approximately 50 years. Individuals living with obesity are at a greater risk of developing dyslipidemia, type 2 diabetes, various cardiovascular diseases, and some cancers [3,53]. In addition to the direct health care costs (~$6 billion annually in Canada) associated with obesity, there are also indirect costs on the Canadian health care system (~$22 billion annually) due to increased absenteeism, additional disability payments, and reduced productivity [54]. The detrimental health effects and substantial economic burden associated with obesity underscore the need for the development of new strategies to manage this chronic disease. While there has been a large surge in antiobesity medications (eg, glucagon-like peptide-1 receptor agonists) in recent years [55], nonpharmaceutical treatments for obesity are still critical, as these medications may not be accessible (due to high cost), manageable (due to potential side effects), or feasible (due to weight gain after ceasing use) for individuals and do not address the low levels of physical activity and cardiorespiratory fitness in this population [4,5]. Exercise represents a nonpharmacological treatment to reduce body weight and body fat while increasing physical activity levels and cardiorespiratory fitness, though there are a variety of barriers that can limit participation and reduce its effectiveness [17]. Exercise snacks appear to be a viable option to simultaneously increase physical activity and cardiorespiratory fitness levels while breaking up sedentary behavior with the potential to lower cardiometabolic risk in individuals living with obesity.

The proposed RCT will compare feasibility-related (primary) and efficacy-related (secondary) outcomes of a real-world exercise snacks intervention to a stretching or mobility exercise comparator group, which would still receive potential benefits from breaking up sedentary time. While this differs from a traditional “no-exercise” control group, it will allow us to isolate the effect of vigorous-intensity exercise on the outcomes, independent of the effect of breaking sedentary time. Selecting feasibility-related primary outcomes was done to determine whether adults living with obesity could be recruited, retained, and adhere to an exercise snacks intervention before conducting a larger, definitive trial focused on efficacy outcomes. Our group has completed 2 exercise snacks feasibility trials, one in healthy inactive adults [26] and one in inactive adults living with type 2 diabetes [56], with both demonstrating that the interventions were feasible and enjoyable (high enjoyment ratings). The study outlined in this protocol is still important as adults living with obesity experience barriers to physical activity (ie, stigma) that are not traditionally experienced by (or may be different from) the populations from our previous studies [18,19].

In an effort to determine the feasibility of testing procedures and derive effect sizes for a future more definitive trial, secondary outcomes are focused on common cardiometabolic health markers, mainly cardiorespiratory fitness, anthropometric-related outcomes, blood pressure, and fasted plasma metabolic markers. These outcomes will test whether brief bouts of vigorous activity interspersed throughout the day have the potential to improve cardiometabolic health over a 12-week intervention in adults living with obesity. While the total exercise volume in this intervention is small, even modest improvements in many of these outcomes can have meaningful impacts on cardiometabolic health and all-cause mortality risk [16,57-59]. This is supported by large-scale epidemiological data reporting that approximately 4 minutes of wearable device–measured vigorous physical activity per day is associated with a 26% to 30% reduction in all-cause mortality [60] and approximately 36% reduction in risk of developing type 2 diabetes [61], which is in line with the exercise snacks prescription in this protocol of 4 × 1 minute exercise snacks on 5 days per week.

Potential limitations and challenges with the proposed RCT include the relatively short duration of the proposed intervention, difficulties in assessing the intensity of exercise snacks completed in a real world, adherence challenges in the real-world setting, and the dose of exercise snacks. The overall duration of the intervention is relatively short (ie, 12 wk), though we believe that this is an appropriate duration to determine the feasibility of an exercise snacks intervention with behavior change counseling and is in line with the duration of other feasibility trials in this field [26,56,62,63]. There are inherent challenges in assessing the intensity of exercise snacks completed outside of the laboratory setting; however, we believe the steps we have in place during the baseline visits can help mitigate this. For example, during baseline visit 2, participants will be explained on how the Borg CR-10 RPE scale works and will complete familiarization or practice exercise snacks with heart rate measurements to ensure that they are performing them at an appropriate intensity given their randomization. Given the real-world nature of the study, it is possible that participants may struggle to adhere to the intervention, though we believe the theory-based behavior change counseling at baseline and throughout the intervention, as well as our team’s strong track record of conducting exercise snacks interventions in the real world [26,56], will mitigate these potential challenges. Finally, the dose of exercise snacks selected for this study (4 × 1-minute bout per day and ≥20 bouts per week) is built on epidemiological data supporting that approximately 4 minutes per day of wearable device–measured vigorous-intensity physical activity is associated with meaningful reductions in all-cause mortality [60]; however, this dose may need to be optimized in future studies for efficacy-related outcomes.

### Conclusion

Exercise snacks are a potential option for adults living with obesity to increase their physical activity and cardiorespiratory fitness while simultaneously reducing barriers to physical activity and their cardiometabolic risk. This study will provide evidence of the feasibility of exercise snacks in inactive adults living with obesity, as well as preliminary efficacy for their ability to improve health. Given the real-world nature of exercise snacks and the targeted counseling throughout the intervention, this type of intervention could be scaled up in the future, if deemed feasible, for a larger more definitive trial.

## Abbreviations

ENMO: Euclidean Norm Minus One
HOMA-IR: Homeostatic model assessment of insulin resistance
PI: principal investigator
RCT: randomized clinical trial
REDCap: Research Electronic Data Capture
RPE: rating of perceived exertion
UBC: University of British Columbia

## References

[1] Body mass index, overweight or obese, self-reported, adult, age groups (18 years and older). Statistics Canada.

[2] (2016). Obesity in Canada: a whole-of-society approach for a healthier Canada. https://sencanada.ca/content/sen/committee/421/SOCI/Reports/2016-02-25_Revised_report_Obesity_in_Canada_e.pdf.

[3] Apovian CM (2016). Obesity: definition, comorbidities, causes, and burden. Am J Manag Care.

[4] Bastin A, Romain AJ, Marleau J, Baillot A (2019). Health behaviours, intentions and barriers to change among obesity classes I, II and III. Clin Obes.

[5] Churilla JR, Johnson TM, Richardson MR, Williams BD, Rariden BS, Boltz AJ (2018). Mode of physical activity participation by body mass index: 2015 Behavioural Risk Factor Surveillance System. Res Sports Med.

[6] O’Donoghue G, Blake C, Cunningham C, Lennon O, Perrotta C (2021). What exercise prescription is optimal to improve body composition and cardiorespiratory fitness in adults living with obesity? A network meta-analysis. Obes Rev.

[7] Pazzianotto-Forti EM, Moreno MA, Plater E, Baruki SBS, Rasera-Junior I, Reid WD (2020). Impact of physical training programs on physical fitness in people with class II and III obesity: a systematic review and meta-analysis. Phys Ther.

[8] van Baak MA, Pramono A, Battista F (2021). Effect of different types of regular exercise on physical fitness in adults with overweight or obesity: systematic review and meta-analyses. Obes Rev.

[9] Pescatello LS, Buchner DM, Jakicic JM (2019). Physical activity to prevent and treat hypertension: a systematic review. Med Sci Sports Exerc.

[10] Liu Y, Wang X, Fang Z (2024). Evaluating the impact of exercise on intermediate disease markers in overweight and obese individuals through a network meta-analysis of randomized controlled trials. Sci Rep.

[11] Mandal AK, Rana T, Shrestha S (2025). Effectiveness of aerobic exercise in reducing blood pressure among obese adults: systematic review and meta-analysis. J Hypertens.

[12] Armstrong A, Jungbluth Rodriguez K, Sabag A (2022). Effect of aerobic exercise on waist circumference in adults with overweight or obesity: a systematic review and meta-analysis. Obes Rev.

[13] Bellicha A, van Baak MA, Battista F (2021). Effect of exercise training on weight loss, body composition changes, and weight maintenance in adults with overweight or obesity: an overview of 12 systematic reviews and 149 studies. Obes Rev.

[14] Jayedi A, Soltani S, Emadi A, Zargar MS, Najafi A (2024). Aerobic exercise and weight loss in adults: a systematic review and dose-response meta-analysis. JAMA Netw Open.

[15] Ross R, Blair SN, Arena R (2016). Importance of assessing cardiorespiratory fitness in clinical practice: a case for fitness as a clinical vital sign: a scientific statement from the American Heart Association. Circulation.

[16] Kodama S, Saito K, Tanaka S (2009). Cardiorespiratory fitness as a quantitative predictor of all-cause mortality and cardiovascular events in healthy men and women: a meta-analysis. JAMA.

[17] Baillot A, Chenail S, Barros Polita N (2021). Physical activity motives, barriers, and preferences in people with obesity: a systematic review. PLoS One.

[18] Bevan N, O’Brien KS, Lin CY (2021). The relationship between weight stigma, physical appearance concerns, and enjoyment and tendency to avoid physical activity and sport. Int J Environ Res Public Health.

[19] Vartanian LR, Shaprow JG (2008). Effects of weight stigma on exercise motivation and behavior: a preliminary investigation among college-aged females. J Health Psychol.

[20] Islam H, Gibala MJ, Little JP (2022). Exercise snacks: a novel strategy to improve cardiometabolic health. Exerc Sport Sci Rev.

[21] Weston KL, Little JP, Weston M (2025). Application of exercise snacks across youth, adult and clinical populations: a scoping review. Sports Med Open.

[22] Caldwell HG, Coombs GB, Rafiei H, Ainslie PN, Little JP (2021). Hourly staircase sprinting exercise “snacks” improve femoral artery shear patterns but not flow-mediated dilation or cerebrovascular regulation: a pilot study. Appl Physiol Nutr Metab.

[23] Jenkins EM, Nairn LN, Skelly LE, Little JP, Gibala MJ (2019). Do stair climbing exercise “snacks” improve cardiorespiratory fitness?. Appl Physiol Nutr Metab.

[24] Little JP, Langley J, Lee M (2019). Sprint exercise snacks: a novel approach to increase aerobic fitness. Eur J Appl Physiol.

[25] Rafiei H, Omidian K, Myette-Côté É, Little JP (2021). Metabolic effect of breaking up prolonged sitting with stair climbing exercise snacks. Med Sci Sports Exerc.

[26] Babir FJ, Islam H, McCreary S (2025). Technology-enabled exercise “snacks” are feasible to perform in a real-world setting: a randomized controlled trial. Scand J Med Sci Sports.

[27] Teare MD, Dimairo M, Shephard N, Hayman A, Whitehead A, Walters SJ (2014). Sample size requirements to estimate key design parameters from external pilot randomised controlled trials: a simulation study. Trials.

[28] Billingham SAM, Whitehead AL, Julious SA (2013). An audit of sample sizes for pilot and feasibility trials being undertaken in the United Kingdom registered in the United Kingdom Clinical Research Network database. BMC Med Res Methodol.

[29] Harris PA, Taylor R, Minor BL (2019). The REDCap Consortium: building an international community of software platform partners. J Biomed Inform.

[30] Harris PA, Taylor R, Thielke R, Payne J, Gonzalez N, Conde JG (2009). Research electronic data capture (REDCap)—a metadata-driven methodology and workflow process for providing translational research informatics support. J Biomed Inform.

[31] Bull FC, Al-Ansari SS, Biddle S (2020). World Health Organization 2020 guidelines on physical activity and sedentary behaviour. Br J Sports Med.

[32] Ross R, Chaput JP, Giangregorio LM (2020). Canadian 24-Hour Movement Guidelines for adults aged 18-64 years and adults aged 65 years or older: an integration of physical activity, sedentary behaviour, and sleep. Appl Physiol Nutr Metab.

[33] Borg GA (1982). Psychophysical bases of perceived exertion. Med Sci Sports Exerc.

[34] Stanley DM, Williams S, Cumming J (2009). Preliminary validation of a single-item measure of exercise enjoyment: the exercise enjoyment scale. J Sport Exerc Psychol.

[35] Döbler A, Herbeck Belnap B, Pollmann H, Farin E, Raspe H, Mittag O (2018). Telephone-delivered lifestyle support with action planning and motivational interviewing techniques to improve rehabilitation outcomes. Rehabil Psychol.

[36] Arkkukangas M, Söderlund A, Eriksson S, Johansson AC (2018). One-year adherence to the Otago Exercise Program with or without motivational interviewing in community-dwelling older adults. J Aging Phys Act.

[37] MacPherson MM, Dineen TE, Cranston KD, Jung ME (2020). Identifying behaviour change techniques and motivational interviewing techniques in Small Steps for Big Changes: a community-based program for adults at risk for type 2 diabetes. Can J Diabetes.

[38] do Valle Nascimento TMR, Resnicow K, Nery M (2017). A pilot study of a Community Health Agent-led type 2 diabetes self-management program using Motivational Interviewing-based approaches in a public primary care center in São Paulo, Brazil. BMC Health Serv Res.

[39] Maxwell-Smith C, Hince D, Cohen PA (2019). A randomized controlled trial of WATAAP to promote physical activity in colorectal and endometrial cancer survivors. Psychooncology.

[40] Pollock ML, Bohannon RL, Cooper KH (1976). A comparative analysis of four protocols for maximal treadmill stress testing. Am Heart J.

[41] Pollock ML, Foster C, Schmidt D, Hellman C, Linnerud AC, Ward A (1982). Comparative analysis of physiologic responses to three different maximal graded exercise test protocols in healthy women. Am Heart J.

[42] Balke B, Ware RW (1959). An experimental study of physical fitness of Air Force personnel. U S Armed Forces Med J.

[43] Kokkinos P, Kaminsky LA, Arena R, Zhang J, Myers J (2017). New generalized equation for predicting maximal oxygen uptake (from the Fitness Registry and the Importance of Exercise National Database). Am J Cardiol.

[44] Matthews DR, Hosker JP, Rudenski AS, Naylor BA, Treacher DF, Turner RC (1985). Homeostasis model assessment: insulin resistance and beta-cell function from fasting plasma glucose and insulin concentrations in man. Diabetologia.

[45] van Hees VT, Fang Z, Langford J (2014). Autocalibration of accelerometer data for free-living physical activity assessment using local gravity and temperature: an evaluation on four continents. J Appl Physiol (1985).

[46] Migueles JH, Rowlands AV, Huber F, Sabia S, van Hees VT (2019). GGIR: a research community–driven open source R package for generating physical activity and sleep outcomes from multi-day raw accelerometer data. J Meas Phys Behav.

[47] Rowlands AV, Edwardson CL, Davies MJ, Khunti K, Harrington DM, Yates T (2018). Beyond cut points: accelerometer metrics that capture the physical activity profile. Med Sci Sports Exerc.

[48] Rowlands AV, Dawkins NP, Maylor B (2019). Enhancing the value of accelerometer-assessed physical activity: meaningful visual comparisons of data-driven translational accelerometer metrics. Sports Med Open.

[49] Edwardson CL, Winkler EAH, Bodicoat DH (2017). Considerations when using the activPAL monitor in field-based research with adult populations. J Sport Health Sci.

[50] Coffman CJ, Edelman D, Woolson RF (2016). To condition or not condition? Analysing “change” in longitudinal randomised controlled trials. BMJ Open.

[51] Efficacy of exercise snacks in real-world settings in individuals living with obesity. Open Science Framework (OSF).

[52] NCD Risk Factor Collaboration (NCD-RisC) (2024). Worldwide trends in underweight and obesity from 1990 to 2022: a pooled analysis of 3663 population-representative studies with 222 million children, adolescents, and adults. The Lancet.

[53] Jensen MD, Ryan DH, Apovian CM (2014). 2013 AHA/ACC/TOS guideline for the management of overweight and obesity in adults: a report of the American College of Cardiology/American Heart Association Task Force on Practice Guidelines and The Obesity Society. J Am Coll Cardiol.

[54] Chen F, Sapra T, Natale Z, Dall TM, Patton I, Sockalingam S (2025). Modeling the cost of inaction in treating obesity in Canada. BMC Public Health.

[55] Yeo YH, Rezaie A, Hsieh TYJ (2024). Shifting trends in the indication of glucagon-like peptide-1 receptor agonist prescriptions: a nationwide analysis. Ann Intern Med.

[56] Babir FJ, Marcotte-Chénard A, Sandilands RE (2026). Exercise snacks are feasible to perform in the real world and improve physical capacity for adults living with non-insulin treated type 2 diabetes: a randomized trial. medRxiv.

[57] Lewington S, Clarke R, Qizilbash N, Peto R, Collins R, Prospective Studies Collaboration (2002). Age-specific relevance of usual blood pressure to vascular mortality: a meta-analysis of individual data for one million adults in 61 prospective studies. Lancet.

[58] de Koning L, Merchant AT, Pogue J, Anand SS (2007). Waist circumference and waist-to-hip ratio as predictors of cardiovascular events: meta-regression analysis of prospective studies. Eur Heart J.

[59] Yi SW, Park S, Lee YH, Park HJ, Balkau B, Yi JJ (2017). Association between fasting glucose and all-cause mortality according to sex and age: a prospective cohort study. Sci Rep.

[60] Stamatakis E, Ahmadi MN, Gill JMR (2022). Association of wearable device-measured vigorous intermittent lifestyle physical activity with mortality. Nat Med.

[61] Chong KH, Ahmadi MN, Biswas RK (2026). Dose-response associations of intermittent lifestyle physical activity micropatterns and incident type 2 diabetes. Diabetes Care.

[62] Pang B, Moullin JC, Thompson C (2025). Testing a vigorous intermittent lifestyle physical activity intervention in adults transitioning to retirement: a pilot randomised controlled trial. Age Ageing.

[63] Daley AJ, Griffin RA, Sanders JP (2025). Snacktivity^TM^ to promote physical activity in primary care, community health and public health settings: a feasibility randomised controlled trial. Int J Behav Med.

